# Ocular Convergence Deficits in Schizophrenia

**DOI:** 10.3389/fpsyt.2012.00086

**Published:** 2012-10-17

**Authors:** Mark S. Bolding, Adrienne C. Lahti, Timothy J. Gawne, Kristine B. Hopkins, Demet Gurler, Paul D. Gamlin

**Affiliations:** ^1^Department of Vision Sciences, University of Alabama at BirminghamBirmingham, AL, USA; ^2^Department of Psychiatry and Behavioral Neurobiology, University of Alabama at BirminghamBirmingham, AL, USA; ^3^Department of Optometry, University of Alabama at BirminghamBirmingham, AL, USA

**Keywords:** convergence insufficiency, schizophrenia, vergence, oculomotor, endophenotype, eye movement dysfunction

## Abstract

Individuals with schizophrenia (SZ) have been reported to exhibit a higher prevalence of convergence insufficiency (CI) than the “normal” adult population. The purpose of this study was to determine if individuals with SZ exhibit clinical signs of CI and to determine if the Convergence Insufficiency Symptom Survey (CISS) is an effective instrument for identifying CI in this population. Twenty participants with SZ and 20 healthy controls (HC) completed the study. The prevalence of CI (15%) in the SZ group was slightly higher than reported norms, but the difference was not significant. The SZ group had significantly higher scores on the CISS than the HC group, but the CISS scores did not correlate with clinical measures of CI in individuals with SZ. The only exception was that SZ patients had a significantly reduced fusional reserve as determined by Sheard’s criteria. Further study is needed to determine why individuals with SZ reported symptoms associated with CI even though clinical measures did not support this diagnosis.

## Introduction

Schizophrenia (SZ) is a complex hereditary mental illness that is characterized by positive, negative, and cognitive symptoms. The diagnosis is based solely on relatively subjective evaluation of symptoms, and no objective assessment is currently available (Tamminga et al., 2010). The etiology and pathophysiology of the disorder remain poorly understood, and little progress has been made in developing novel treatments (Insel, 2009).

When studying complex genetic disorders a standard approach is to search for endophenotypes, as these could provide a bridge between complex clinical phenotypes and specific genotypes (Ritsner, 2009). They are defined as discrete, heritable, genetically determined variables that are not easily discerned by clinical assessment but discoverable by a “biochemical test or microscopic examination” (Lenox et al., 2002; Gottesman and Gould, 2003).

Abnormalities in the visual system, specifically eye movement dysfunctions, have long been considered as possible endophenotypes in SZ (Turetsky et al., 2007; Silverstein and Keane, 2011). Of these, smooth pursuit and saccadic eye movements deficits are the most replicated findings (Levy et al., 1994; Trillenberg et al., 2004; Zanelli et al., 2005; O’Driscoll and Callahan, 2008; Rommelse et al., 2008). These deficits persist even if patients are treated with antipsychotic medications (Levy et al., 1983; Ulrich and Veena, 2003) and are also found in unaffected relatives of patients with SZ (Ross et al., 2002; Kathmann et al., 2003; Calkins et al., 2003; Zanelli et al., 2009).

Because the neural substrates of vergence overlap with those of smooth pursuit (Gamlin, 2002; Lynch and Tian, 2006), they are a potentially important class of eye movements to investigate in patients with SZ. Vergence, a binocular disconjugate eye movement that aligns the eyes to targets at specific depths, became necessary with the development of binocular vision in foveate primates and is thought to be cognitively demanding. Levin et al. (1982) reported a higher rate of intrusive saccades in vergence tracking in patients with SZ than in healthy controls (HC). In addition, in a prospective study of high-risk individuals, Schiffman et al. (2006) reported a higher rate of binocular alignment abnormalities in individuals who later developed SZ, suggesting a possible pre-morbid relationship between oculomotor deficits and SZ. These results suggest that there might be a link between vergence eye movements and SZ, but compared to the research on smooth pursuit and saccadic eye movements relatively little is known.

One common deficit of vergence eye movements is convergence insufficiency (CI). CI is a deficit in the ability to converge the eyes to near targets. Consequences of CI include headaches, blurry and double vision, and difficulty reading or performing near work. In contrast to tests of smooth pursuit or antisaccades, CI is routinely measured in clinical exams. Several studies suggest CI is present in individuals with SZ (Flach et al., 1992; Hobart et al., 1999; Chan et al., 2009). However, these reports were based on subjective judgments of CI. Objective criteria such as measures of eye position, near point of convergence (NPC), phoria, or positive fusion vergence ranges were not assessed. To date, there have been no definitive quantitative studies of CI in individuals with SZ.

The current study was designed to measure objective clinical signs of CI in individuals with SZ and matched HC. A secondary goal of the study was to test the validity of the Convergence Insufficiency Symptom Survey (CISS) for detecting symptoms associated with CI in patients with SZ. We included 20 stable patients with SZ and 20 matched HC to test the following hypotheses: (1) compared to HC, patients with SZ will show an increased rate of CI, as measured using standard clinical tests and, (2) increased rate of CI in patients will be correlated with increased numbers of symptoms reported on the CISS.

## Materials and Methods

Twenty-four subjects with SZ and schizoaffective disorder were recruited from the outpatient psychiatry clinic at The University of Alabama at Birmingham (UAB) to participate in this study. Twenty-three HC, matched on age, gender, ethnicity, and parental occupation, were recruited by advertisement in flyers and the university’s newspaper. Exclusion criteria were major medical conditions, substance abuse within 6 months of enrollment, previous serious head injury, a neurological disorder, loss of consciousness for more than 2 min, visual acuity of less than 20/40 in either eye, more than two lines of difference in visual acuity between the eyes, or lack of stereopsis. The study was approved by the Institutional Review Board of the UAB, and all subjects gave written informed consent. Before signing consent, each SZ subject completed an Evaluation to Sign Consent Form.

Diagnoses were established using subjects’ medical records and the Diagnostic Interview for Genetic Studies (DIGS; Nurnberger et al., 1994). Cognitive function was characterized by the Repeatable Battery for the Assessment of Neuropsychological Status (RBANS; Randolph et al., 1998; Gold et al., 1999; Wilk et al., 2002). The Brief Psychiatric Rating Scale (BPRS; Overall and Gorham, 1962) and its positive and negative subscales were used to assess symptom severity.

Each subject was examined by the same physician who was blind to the psychiatric diagnosis. Three participants (SZ = 1) were excluded during vision screening and four (SZ = 3) withdrew or were lost to follow up. Forty participants, 20 SZ and 20 HC, completed the study and were included in the final analyses.

### Clinical assessment

All visual measures were obtained while subjects were stable on their medication regimen. Distance visual acuity was measured in each eye with a projected Snellen chart at 20 feet. Near visual acuity was screened in each eye with a 20/30 isolated line of letters. Binocular vision testing included fixation disparity (Saladin card), ocular alignment with cover test at distance and near, NPC break and recovery, positive fusional vergences (PFV) at near break and recovery (prism bar), stereo acuity (Randot Stereo), accommodative amplitudes (push-up) for non-presbyopes, and distance and near auto-refraction.

Near point of convergence was measured three times with the Astron International Accommodative Rule (ACR/21; Gulden Ophthalmics, Elkins Park, PA, USA) using a printed fixation target with a single column of 20/30 letters. The target was initially positioned at eye level, 40 cm from the participant’s bridge and then moved toward the subject’s eyes until either the subject reported seeing the target doubled or the examiner noticed a break in fusion (one eye drifted off target). If the subject was not able to regain fusion, the distance from the eyes where fusion was lost was noted as the NPC break point. The target was then pulled away from the participant’s eyes until fusion was regained and this distance was documented as the recovery point. The three NPC measures were averaged for analysis.

Positive fusional vergences were measured three times with a horizontal prism bar (Gulden B-16 horizontal prism bar) while the subject fixated a hand-held fixation target (Gulden fixation stick #15302) with a single column of 20/30 letters held at eye level at a distance of 40 cm. Base out prism was introduced in gradient steps on the prism bar while the patient was asked to report if the target became blurred or double. The prism amounts where blur and then double vision were reported were recorded as the blur and break values respectively. Once fusion had been lost, the prism amount was decreased until fusion was recovered and this prism amount was recorded as the recovery value. If the examiner noticed a loss of fusion without the report of double vision, this value was also recorded as the break value. Three fusional vergence measures were taken and the results were averaged.

The CISS was administered to each subject at the beginning of the visit and again at the end of the exam. The survey contains 15 questions regarding how the subject’s eyes feel when reading or doing close work. The examiner presented questions verbally and participants were asked to respond with one of the phrases/words printed on a hand-held card (never, infrequently, sometimes, fairly often, or always). Each response was scored between 0 (never) and 4 (always) to give a final symptom score range of 0–60. For analysis, the two symptom scores were averaged.

### Classification of CI

Participants were categorized as having CI if they met the following three criteria: phoria at near of 4 prism diopters or more greater exophoria than at distance; a NPC break greater than 5.5 cm, and near PFV less than 15 prism diopters or failing Sheard’s criteria PFV less than twice the near phoria, discussed below. Participants were classified as symptomatic if the CISS average was 21 or greater. Results for the participants with SZ were compared to published norms (Daum, 1988; Rouse et al., 2004, 2009). Statistics were calculated in *R* (the R project for statistical computing). Group means were compared using *t*-tests (two-tailed, Welch correction for unequal variance) unless otherwise noted.

### Sheard’s criteria

Sheard’s criteria states that patients should have “a fusional vergence reserve at least twice the magnitude of (their) heterophoria” (Sheard, 1938). Proposed by Sheard in the 1930s, this rule is frequently used by optometrists to prescribe prism. As yet, there have been few studies to test its validity. The studies that have been done generally support the validity of Sheard’s criteria, but suggest that it may not detect some patients who are symptomatic (Daum et al., 1989). Studies also suggest that this test is better at predicting discomfort in exophores than in esophores (Sheedy and Saladin, 1978).

## Results

### Group matching

There were no significant differences in age, race, gender, smoking status, or parental socioeconomic status between the SZ and HC participant groups (Table 1).

**Table 1:** **Demographics and clinical measures^a^**.

Characteristic	HC (*n* = 20)^f^	SZ (*n* = 20)	*t*/χ^2^	*p*-value
Age, years	36.3 ± 11.3	39.0 ± 11.4	0.75	0.46
Gender, F/M	8/12	9/11	0.10	0.75
Ethnicity, AA/C^b^	10/10	14/6	0.94	0.33
Parental SES^c^	6.7 ± 5.1	6.8 ± 5.0	0.07	0.94
**RBANS^d^**				
Total index	87.2 ± 12.5	73.7 ± 10.2	6.16	0.002
Immediate memory	88.6 ± 15.3	77.5 ± 12.3	6.43	0.03
Visuospatial	79.9 ± 15.7	79.9 ± 15.7	2.31	0.69
Language	95.5 ± 14.4	87.3 ± 13.7	2.95	0.11
Attention	96.4 ± 20.8	82.3 ± 12.9	4.58	0.03
Delayed memory	91.8 ± 8.4	72.5 ± 20.7	5.38	0.001
**BPRS^e^**				
Total	–	29.2 ± 6.8	–	–
Positive	–	4.5 ± 2.6	–	–
Negative	–	4.3 ± 2.0	–	–

*χ^2^ includes Yate’s correction*.

*^a^Mean ± SD unless indicated otherwise; SZ, schizophrenia; HC, healthy control*.

*^b^AA, African American; C, Caucasian*.

*^c^Socioeconomic Status; Ranks determined from Diagnostic Interview for Genetic Studies (1–18 scale); higher rank (lower numerical value) corresponds to higher socioeconomic status; information not available for one SZ*.

*^d^Repeatable Battery for the Assessment of Neuropsychological Status; data not available for two SZ and two HC*.

*^e^Brief Psychiatric Rating Scale (1–7 scale); positive (conceptual disorganization, hallucinatory behavior, and unusual thought content); negative (emotional withdrawal, motor retardation, and blunted affect); data not available for two SZ*.

*^f^18 SZ were treated with second generation antipsychotics and two SZ were not taking antipsychotics*.

### Convergence insufficiency symptom survey

**Figure 1:**
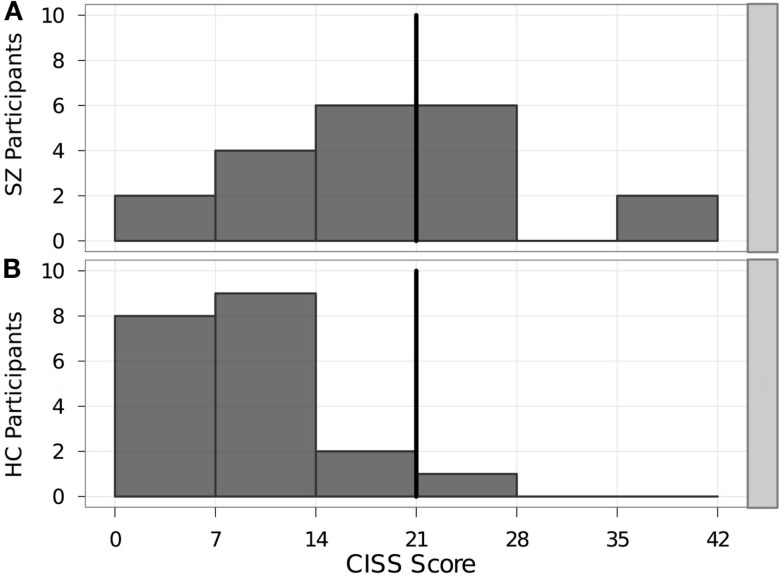
**Distribution of mean CISS scores in each participant group**. Bold vertical line indicates the cutoff score of 21. Individuals scoring ≥ 21 on the CISS were considered as having symptoms consistent with CI. **(A)** SZ, schizophrenia patients. **(B)** HC, healthy controls. CISS, Convergence Insufficiency Symptom Survey.

Eight SZ and one HC met or exceeded the cutoff score of 21 on the CISS (Figure 1). The mean CISS score (18.6 ± 9.9) for the SZ group was significantly higher than that of the HC group (9.9 ± 5.5). In addition, the SZ group exhibited significantly (*p* = 0.002) higher CISS scores than those reported for subjects with normal binocular vision and stereopsis, regardless of whether they also had CI (11.0 ± 8.2; Rouse et al., 2004).

### Clinical measures

#### CI criteria measures

Three of the SZ participants and one of the HC participants met all three of the diagnostic criteria and were classified as convergence insufficient (Table 2). Seven SZ and six HC had a receded NPC. The mean NPC for the SZ and HC groups were 5.5 and 4.4 cm respectively, which was not a significant difference (*p* = 0.207). Eight SZ and nine HC met the cover test criteria for CI. The mean phoria difference in the cover test for the SZ group was −3.95 prism diopters while the HC group was −2.35 prism diopters. The phoria differences between groups were also not significant (*p* = 0.626). Seven SZ and one HC failed Sheard’s criteria. This difference was significant (*p* = 0.022). With our cutoff values, the chi-square test for differences between NPC and phoria classifications were not significant (*p* = 0.736, *p* = 0.749 respectively).

**Table 2:** **Convergence insufficiency symptom survey scores and CI clinical measures, between group comparisons^a^**.

Criterion measure	HC (*N* = 20)	SZ (*N* = 20)	*t*/χ^2^	*p*-Value
**CISS**	9.9 ± 5.5	18.6 ± 9.9	*t* = 3.43	0.001
<21	19	12		
≥21	1	8		
**CI**				
CI negative	19	17	Fischer	0.60
CI positive	1	3	Exact test	
**NPC criteria**				
≤5.5 cm	14	13	χ^2^ = 0.11	0.73
>5.5 cm	6	7		
**Sheard’s criteria**				
True	1	7	Fischer	0.02
False	19	13	Exact test	
**Phoria criteria**				
True	9	8	χ^2^ = 0.10	0.75
False	11	12		
**NPC break** (cm)	4.4 ± 2.37	5.5 ± 2.95	*t* = 1.30	0.20
**NPC recover** (cm)	6.88 ± 3.28	8.45 ± 3.02	*t* = 1.57	0.12
**Cover test** (PD^b^)	−2.35 ± 3.45	−3.95 ± 6.76	*t* = 0.94	0.35

*χ^2^ statistic includes Yate’s correction. Fischer’s exact test was used when there were less than five cases in a cell in a given contingency table*.

*^a^Mean ± SD unless indicated otherwise; SZ, schizophrenia; HC, healthy control*.

*^b^PD prism diopters, near phoria – distance phoria*.

#### Interrelation of CI clinical measures

Highly significant correlations were found between all of the measures used to form the CI criteria (all *p* < 0.0005) in the entire cohort, this was also true when only SZ participants (all *p* < 0.05) or HC participants (all *p* < 0.05) were included in the analysis.

#### Relationship of CISS scores and clinical measures in the SZ group

Eight SZ participants exceeded the cutoff score of 21 on the CISS (Table 2). However, there were no significant differences in NPC break or recovery, vergence break or recovery, distance or near phoria, phoria differences at near vs. distance, stereo acuity, or fixation disparity between the SZ participants who scored at or above the CISS cutoff and those who scored below the cutoff.

The mean CISS score for the SZ participants with CI did not significantly differ from those without (with CI: 15.83; without CI: 19.06; *p* > 0.05). The three SZ participants who met the clinical criteria for CI all scored below 21 on the CISS (Table 3). There was no significant relationship between the CISS scores and any clinical measure of CI.

**Table 3:** **SZ CISS scores and CISS classification frequency by CI diagnosis^a^**.

CISS scores	CI positive	CI negative
Mean	15.83 ± 1.25	19.06 ± 10.72
*n* < 21	3	9
*n* ≥ 21	0	8

*^a^Mean ± SD unless indicated otherwise; SZ, schizophrenia; CISS, Convergence Insufficiency Symptom Survey*.

#### Relationship of CISS and clinical CI measures with psychometric tests

As expected, SZ and HC had significantly different scores on the RBANS (*p* = 0.002). In the SZ group there was no significant difference between those who scored 21 or above on the CI and those who did not on the BPRS total, BPRS negative or positive subscales scores, the RBANS total or its subscales scores. There was no significant correlation between the CISS mean scores and any of the psychometric total scores or subscale scores in either group.

There was no significant difference between SZ with CI and those without and any of the psychometric total scores or subscale scores. There was no significant correlation between the NPC break, phoria measures, or vergence ductions and any of the psychometric total scores or sub scores.

## Discussion

In this study, we compared objective clinical measures of CI with the CISS subjective self-reports of CI symptoms in a group of stable patients with SZ, and matched HC. Contrary to our initial hypotheses, we found that patients with SZ did not exhibit CI more frequently than HC, although their CISS-based self-report measures suggested the opposite.

The two eye movement deficits that have been most extensively studied in SZ, smooth pursuit gain and antisaccade error rate, are neither specific to the disorder nor present in all patients with SZ (Holzman, 2000; Hutton et al., 2004; Levy et al., 2004; Donohoe et al., 2006). They do however provide insights into the neuropathology of SZ and have proved to be useful endophenotypes by reducing heterogeneity in genetic studies of SZ (Calkins et al., 2008). Because the neural substrates of vergence eye movements overlap those of smooth pursuit (Lynch and Tian, 2006), they are a particularly important class of eye movements to investigate in patients with SZ. In addition, if in fact abnormal vergence eye movements are associated with SZ then, because CI is routinely measured in clinical exams, large-scale characterization of vergence eye movement dysfunction in SZ would be possible.

Only one objective measure of CI was found to be significantly different between the groups: SZ met Sheard’s criteria more frequently than HC. This difference may indicate that, even though the patients have normal ranges of convergence, they have less reserve capacity and may experience binocular vision problems when fatigued. This is potentially clinically relevant as oculomotor dysfunction related to binocular vision problems such as CI, could negatively impact the ability of a patient to achieve optimal function (Reding and Potes, 1988; Groswasser et al., 1990). We speculate that if vision therapy for CI could enhance the reserve capacity for convergence, conceivably this could assist in cognitive remediation in SZ and thus help alleviate the greatest contributor to poor functional outcomes (Wykes et al., 2011). Additional research on the effects of vision therapy on convergence reserve capacity, and its interactions with SZ, may be called for.

The finding that SZ patients do not exhibit CI does not necessarily rule out all problems with vergence eye movements. Since CI is a measure of static alignment of the two eyes in near space, it does not require the dynamic sensorimotor transformation required for accomplishing rapid vergence eye movement or tracking-in-depth tasks. The eye movement abnormalities in SZ patients have previously been identified in tasks that require integrating visual information across space and time with a motor output (Chen et al., 2011). Consistent with this, in a preliminary study, we have observed that SZ patients have lowered dynamic vergence tracking gain compared to controls (Bolding et al., 2012).

Interestingly, we found no correlation between the CI clinical measures and CISS ratings in the SZ group. Of the three SZ participants diagnosed with CI, none had a CISS score greater than or equal to 21. In the general population, the CISS has shown good sensitivity (97.8%) for identifying symptomatic CI individuals (Rouse et al., 2004), however this was not true when we applied this to our cohort of SZ patients. Eight participants in the SZ group scored 21 or greater on the CISS although they did not have objective findings consistent with CI. It is possible that patients did not understand the CISS questions. If this were the case, we would expect the CISS scores to negatively correlate with the RBANS scores. However, the CISS scores did not correlate with the RBANS scores or any of its subscales, which indicates that the unexpected high number of false positives is not directly contributable to cognitive deficits. More likely, the large number of false positives on the CISS in the SZ group may suggest that SZ participants experience some visual difficulties related to CI, possibly some dynamic aspect of vergence eye movements not measured by the clinical tests of CI.

In conclusion, contrary to our initial hypothesis, patients with SZ did not exhibit a higher prevalence of CI, although there was evidence for a reduced convergence reserve capacity as defined by Sheard’s criteria. However, 40% of them did report subjective symptoms associated with CI as reported by the CISS. Based on our results, we propose that current clinical CI testing is not an adequate tool to assess prevalence of binocular vision deficits in individuals with SZ. Further study is needed to determine why individuals with SZ are reporting symptoms associated with CI even though standard static clinical measures do not reveal a deficit. The development of simple and robust clinical tests of the dynamics of vergence eye movements, and their application as a research tool in SZ, may prove to be of particular utility.

## Authors Contribution

Mark S. Bolding, Kristine B. Hopkins, Paul D. Gamlin, and Adrienne C. Lahti designed the experiments. Mark S. Bolding and Kristine B. Hopkins performed the experiments and analyzed the data. Mark S. Bolding, Timothy J. Gawne, Kristine B. Hopkins, Paul D. Gamlin, and Adrienne C. Lahti wrote the paper.

## Conflict of Interest Statement

The authors declare that the research was conducted in the absence of any commercial or financial relationships that could be construed as a potential conflict of interest.
